# Characteristics of Mobile Health Platforms for Depression and Anxiety: Content Analysis Through a Systematic Review of the Literature and Systematic Search of Two App Stores

**DOI:** 10.2196/27388

**Published:** 2022-02-04

**Authors:** Qiao Ying Leong, Shreya Sridhar, Agata Blasiak, Xavier Tadeo, GeckHong Yeo, Alexandria Remus, Dean Ho

**Affiliations:** 1 N.1 Institute for Health National University of Singapore Singapore Singapore; 2 The Institute for Digital Medicine (WisDM) Yong Loo Lin School of Medicine National University of Singapore Singapore Singapore; 3 Department of Biomedical Engineering NUS Engineering National University of Singapore Singapore Singapore; 4 Department of Pharmacology Yong Loo Lin School of Medicine National University of Singapore Singapore Singapore; 5 Health District @ Queenstown Singapore Singapore

**Keywords:** mHealth, digital medicine, anxiety, depression, systematic review, mental health conditions, mobile phone

## Abstract

**Background:**

Mobile health (mHealth) platforms show promise in the management of mental health conditions such as anxiety and depression. This has resulted in an abundance of mHealth platforms available for research or commercial use.

**Objective:**

The objective of this review is to characterize the current state of mHealth platforms designed for anxiety or depression that are available for research, commercial use, or both.

**Methods:**

A systematic review was conducted using a two-pronged approach: searching relevant literature with prespecified search terms to identify platforms in published research and simultaneously searching 2 major app stores—Google Play Store and Apple App Store—to identify commercially available platforms. Key characteristics of the mHealth platforms were synthesized, such as platform name, targeted condition, targeted group, purpose, technology type, intervention type, commercial availability, and regulatory information.

**Results:**

The literature and app store searches yielded 169 and 179 mHealth platforms, respectively. Most platforms developed for research purposes were designed for depression (116/169, 68.6%), whereas the app store search reported a higher number of platforms developed for anxiety (Android: 58/179, 32.4%; iOS: 27/179, 15.1%). The most common purpose of platforms in both searches was treatment (literature search: 122/169, 72.2%; app store search: 129/179, 72.1%). With regard to the types of intervention, cognitive behavioral therapy and referral to care or counseling emerged as the most popular options offered by the platforms identified in the literature and app store searches, respectively. Most platforms from both searches did not have a specific target age group. In addition, most platforms found in app stores lacked clinical and real-world evidence, and a small number of platforms found in the published research were available commercially.

**Conclusions:**

A considerable number of mHealth platforms designed for anxiety or depression are available for research, commercial use, or both. The characteristics of these mHealth platforms greatly vary. Future efforts should focus on assessing the quality—utility, safety, and effectiveness—of the existing platforms and providing developers, from both commercial and research sectors, a reporting guideline for their platform description and a regulatory framework to facilitate the development, validation, and deployment of effective mHealth platforms.

## Introduction

Mobile health (mHealth) refers to medical, health care, and public health practices that use mobile computing or technologies such as mobile phones, wireless devices, patient monitoring devices, and web-based platforms [1-3]. With the overarching objective of improving health-related outcomes and awareness, mHealth platforms can either serve as standalone or complementary platforms and offer the potential to cater to the needs of many users, from health care professionals to patients and consumers. The existing infrastructure and technology of mobile devices, such as mobile apps and mobile sensors, make them ideal candidates for offering convenience while addressing clinical concerns and improving health-related outcomes. For example, mobile apps can be installed on a mobile device that allows for the measurement and collection of vital information such as location data and activities. These tracking data are invaluable as they may help inform users about their health status or provide necessary information to health care professionals during their decision-making process [4] to subsequently provide actionable feedback to their patients [5]. In addition, as most of the world’s population already uses mobile devices, it is possible that mHealth interventions may allow greater access to care [6]. According to a digital global report published in October 2020, mobile phones and the internet had a penetration rate of 60% and 67% worldwide, respectively [7], both of which increased by a staggering 1% in just 3 months compared with July 2020 [8]. It is plausible that the COVID-19 pandemic that has driven most of the world into forceful isolation may have contributed to this increment. It is evident that mobile devices are increasingly becoming an indispensable part of our lives and will continue to be so [9]. As such, mHealth may offer a feasible approach for targeting a wide range of health conditions.

In recent years, there has been an increase in the use of mHealth platforms to intervene in the management of mental health conditions [10,11]. As a result, research is emerging on the implementation and clinical outcomes of mHealth platforms [10,11], and preliminary evidence on their efficacy is beginning to surface. Anxiety and depression, which have the highest prevalence among mental health conditions, are at the forefront of these targeted mHealth interventions [12,13]. The convenience facilitated by technology coupled with a higher quality of interventions—evidenced from randomized controlled trials (RCTs) and real-world evidence (RWE) delivered for those with anxiety or depression compared with those delivered for people with general health issues [10,11]—further propels mHealth as an increasingly appealing solution for the prevention and management of anxiety or depression. As a result, a plethora of mHealth platforms for anxiety or depression have been and continue to be developed.

This accelerated proliferation of anxiety or depression mHealth platforms that are readily available makes it difficult for stakeholders to determine not only those that may be useful but also those that may be potentially counterproductive. In addition, the resulting quantity of available platforms does not necessarily correlate with the scale of effectiveness validation. mHealth, as a whole, is still a relatively new field. Subsequently, regulatory policies and standards for the development of these platforms and the claims made by their developers have not been fully established [14,15], adding to the difficulty for stakeholders in identifying appropriate and safe mHealth apps. Accordingly, as the number of mHealth platforms for anxiety or depression continues to grow, it is important for relevant stakeholders, including users, clinicians, researchers, and developers, to understand what is currently available.

A recent review [16] has provided a comprehensive overview of commercially and academically available mental health apps offered in the Spanish language in the United States; however, a similar review of mental health mHealth apps available in the English language, to our knowledge, has not been conducted to date. Therefore, the aim of this review is to systematically identify and amalgamate the characteristics of the currently available English language mHealth platforms for anxiety or depression that have been developed for research, commercial use, or both. The outcome of this review may provide insights into considerations in the development, regulation, implementation, and adoption of future mHealth platforms for anxiety or depression.

## Methods

### Study Design

A cross-sectional study was performed to characterize mHealth platforms developed for depression or anxiety that were available for commercial, research, or both purposes. The mHealth platforms included apps, websites, and web-based software. A 2-pronged approach was used in this study. This included systematically searching relevant literature that described mHealth platforms as interventions developed for research and simultaneously searching 2 major app stores systematically to identify commercially available platforms. The protocol was registered on PROSPERO (International Prospective Register of Systematic Reviews; CRD42020193956) after preliminary searches were conducted. The piloting of inclusion and exclusion criteria and formal screening were conducted after the official PROSPERO registration.

### Identifying Platforms Described in Research Literature

#### Search

The PRISMA (Preferred Reporting Items for Systematic Reviews and Meta-Analyses) guidelines were used and adhered to for the literature search [17]. A systematic search of the databases PubMed, Embase, CINAHL, and PsycINFO was completed on June 23, 2020, from database inception dates. The search string was developed using the population, intervention, comparison, outcome strategy [18] using keywords and search terms combined with Boolean operators (Textbox 1). The search terms were carefully selected so that the homogeneous terms could be used in both the literature and app store searches. The full search strings per database can be found in Multimedia Appendix 1. All searches were performed with no restriction on the publication period or year of release. There were no age or location restrictions on the target population.

**Keywords and search terms used for the formulation of the search string in the literature search.**

**Mental health condition**

- *Mental health, mindfulness, anxiety, depression*

**Platform**

- *mHealth, mobile health, mobile device, mobile application, digital therapeutics, digital intervention, ehealth, smartphone, mobile phone, text message, web based, web application*

#### Selection

After duplicate articles were removed, 2 reviewers (SS and QYL) independently applied the eligibility criteria to titles and abstracts and subsequently to full-text articles using the Covidence software (Veritas Health Innovation) [19]. A third reviewer (AR) resolved any discrepancies. Articles were included if they were published in English, were full-text original journal articles or conference papers, and provided at least one characteristic of interest about an mHealth platform for anxiety or depression.

Articles were excluded if they were reviews (but reference lists were cross-checked for included studies), had no characteristic description of the mHealth platform, did not have the name of the mHealth platform, were not designed specifically for anxiety or depression, described mHealth platforms that targeted physical conditions only, or targeted secondary mental health problems related to a physical condition, health behaviors (eg, smoking cessation, risky drinking, obesity, and exercise), neurodegenerative disorders (eg, dementia), chronic pain, a specific phobia, posttraumatic stress disorder, addictions, subclinical symptoms, or a severe mental illness.

As the primary focus of the review was to characterize mHealth platforms, a formal assessment of the study quality and risk of bias was not performed.

### Identifying Platforms Available Commercially

#### Search

Building on the methodologies by Bender et al [20] and Giunti et al [21], we systematically completed our search of all available platforms from 2 major app stores accessible from Singapore—Google Play Store and Apple App Store—on June 19, 2020. In-depth searches across the Apple App Store were included to identify platforms compatible with iOS or macOS devices. The search terms used were *anxiety*, *depression*, *mental health*, and *mindfulness* (Textbox 1), with no restriction on the app category.

#### Selection

Apps were initially included if the title or description of the app included the terms anxiety or depression. After the removal of duplicates, 2 reviewers (SS and QYL) independently assessed the eligibility of the app using the a priori determined criteria. Any discrepancies were addressed by a third independent reviewer (AR).

The included mHealth platforms comprised apps targeting mental health, specifically anxiety, depression, or both. Apps were excluded if they were no longer available, not available in English, or provided app descriptions that lacked the characteristics of interest.

### Data Coding and Extraction

#### Data Coding

An mHealth platform characteristic coding scheme was developed based on an internally identified mHealth platform’s characteristics of interest. If the mHealth platform characteristics were not clearly described or discrepancies were found during the classification of the mHealth platforms, discussions were held until consensus was reached. The final coding scheme and outcomes are detailed in Table 1.

**Table 1:** The mobile health (mHealth) characteristic coding scheme of the characteristics of interest from the respective searches and the description of each item.

Characteristic	Description	Literature search	App store search
Platform name	The name of the platform that was developed for research, available for commercial use, or both	✓	✓
Targeted condition	The condition the platform was designed for: anxiety, depression, or both	✓	✓
Targeted group	The intended users the platform was designed for	✓	✓
Commercial availability	The commercial availability of the platform in either the Apple App Store, Google Play Store, or both	✓	✓
Purpose of platform	The intended use that the platform was designed for. For example, diagnostics, monitoring, prevention, treatment, education, and support	✓	✓
Type of technology	The mobile modality and operating system in which the mHealth platform was rolled out	✓	✓
Type of intervention	Applicable only for platforms that offer treatment	✓	✓
App store categorization	App store category assigned to the platform by the development team that best describes its main function or subject matter		✓
Additional mHealth characteristics	Additional characteristics reported such as language, ratings, and cost of the platform	✓	✓
Clinical evidence and regulatory information	A secondary search was performed to obtain additional information of the identified platforms which included the availability of clinical evidence in the form of randomized control trials, real-world evidence, and regulatory approval	✓	✓
Other studies that reported the platform	Studies cited in the platform’s article regarding the development, validation, or further evaluation of the platform	✓	
Cited literature	References to academic publications provided in the platform’s app store description		✓

#### Data Extraction

The mHealth platforms and their characteristics of interest from both searches were extracted and recorded in a custom-developed Microsoft Excel template. The collected and assessed data included characteristic information of the mHealth platforms, which were based on the description in the app stores, provided by 2 reviewers (SS and QYL), and any discrepancies were resolved by a third reviewer (AR).

### Statistical Analysis

Descriptive statistical analyses were performed for all the variables. The categorical variables were presented as absolutes and relative frequencies. Statistical analyses were performed using Microsoft Excel (version 16.43).

## Results

### Platforms Available in Literature Search

#### Selection

**Figure 1:**
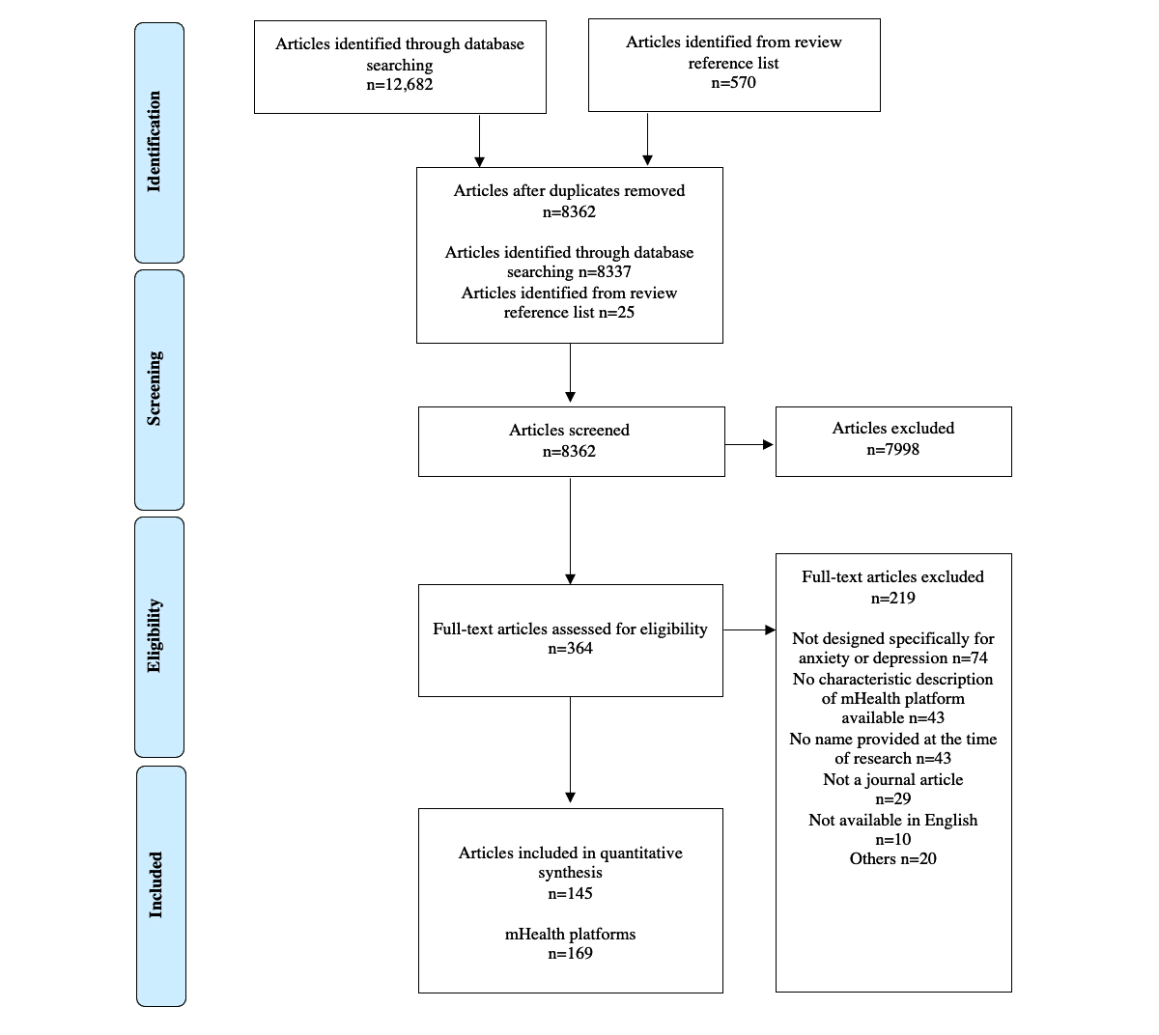
PRISMA (Preferred Reporting Item for Systematic Reviews and Meta-Analyses) flow diagram of search strategy and the finalized number of articles and resulting mHealth platforms included for this review. mHealth: mobile health.

As depicted in Figure 1, after duplicate removal and review of reference list cross-checking, 8362 articles were initially screened. This resulted in the identification of 169 mHealth platforms described in 1.7% (145/8362) of articles (Multimedia Appendix 2 [22-166]). Some articles reported ≥1 platform, whereas some platforms were described in ≥1 article. Examples of platforms found across multiple studies were Deprexis (6/169, 3.6%), MoodGYM (6/169, 3.6%), Happy@Work (4/169, 2.4%), myCompass (4/169, 2.4%), and Partners in Parenting (4/169, 2.4%).

#### Commercial Availability of Apps From the Literature Search

Of the 169 apps identified in the literature search, 8 (4.7%) were available in the app stores. All 8 apps were found in the Google Play Store, whereas a subset (6/8, 75%) was commercially available in the Apple App Store. No platform was reported as a medical device. However, of the 169 platforms, 2 (1.2%)—myStrength and HelpID—were reported as being used in health care settings. myStrength was used as a psychological intervention and had affiliations with health care providers, whereas HelpID was included in health care plans and insurance.

#### Targeted Condition

Of the 169 platforms reported in the articles, 116 (68.6%) targeted depression, 22 (13%) targeted anxiety, and 31 (18.3%) targeted both (Table 2). Approximately 1.8% (3/169) platforms—Situman, TeleCoach, and LifeRhythm—acted as complementary apps to other platforms targeting depression. The roles played by these apps varied, where Situman provided situational awareness to the Moodbuster platform, TeleCoach involved weekly calls to help with adherence to the MoodManager intervention, and LifeRhythm helped in data collection for the DepWatch system.

**Table 2:** Summary of the mobile health (mHealth) platforms characteristics from the literature search (N=169).

mHealth platform characteristics		Platforms, n (%)
**Targeted condition**		
	Anxiety	22 (13)
	Depression	116 (68.6)
	Both	31 (18.3)
**Target group**		
	Children	3 (1.8)
	Young people	15 (8)
	University students	3 (1.8)
	Adults	8 (4.7)
	Older adults	1 (0.6)
	Employees	3 (1.8)
	Migrants	1 (0.6)
	Patient or clinician	2 (1.2)
	Not specified	136 (79.9)
**Purposes**		
	1 only	90 (53.3)
	2	71 (42)
	≥3	8 (4.7)
**Number of languages**		
	1	2 (1.2)
	2	5 (3)
	≥3	4 (2.4)
	Not specified	158 (93.5)

#### Targeted Group

Of the 169 platforms reported in the articles, 135 (79.9%) were not developed for a specific target group. The remaining 20.1% (34/169) targeted 8 different groups: children, young people, university students, adults, older adults, employees, migrants, and patient or clinician communication. Of the 169 apps, 2 (1.2%)—BiP Anxiety and Supporting Our Valued Adolescents—involved ≥1 target group and required the engagement of parents along with their children and adolescents, respectively.

#### Purpose

Approximately half of the platforms were designed with ≥1 purpose (Table 2). Treatment was the most common purpose, with 72.2% (122/169) of the platforms including it (Table 3).

**Table 3:** The primary purpose of mobile health platforms for anxiety or depression identified in the literature search (N=169).

Purpose	Description	Example	Platforms, n (%)
Education	Provide mental health and medication information Teach a variety of skills, including self-management and problem solving Impart lessons through modules	Text-based information, audio or visual materials, and blogs	45 (26.6)
Diagnostic	Identify depression and anxiety symptoms Serve as a screening tool	Psychological questionnaires before use or over time	12 (7.1)
Monitoring	Track self-reported symptoms and medical adherence Collect relevant sensor data such as location and mobility information	Ecological momentary assessment through a mobile app, prompts to rate mood or complete short questionnaires, and data collection through wearable devices and mobile phones	38 (22.5)
Treatment	Deliver psychological interventions through modules Teach a variety of skills such as positive emotion skills, self-management skills, and guided meditation Learn about coping strategies	Cognitive behavioral therapy, problem-solving therapy, gamification, lessons by topic, and homework	122 (72.2)
Prevention	Similar to Treatment but targets individuals who are at risk of anxiety or depression	Text-based information and audio or visual, synchronous, or offline asynchronous activity	29 (16.2)
Support	Offer a decision support system to clinicians Manage care activities Provide access to peer-to-peer support or health care professionals for support	Messaging service, discussion forum, web-based interaction in self-help groups, and web-based directory of bodies offering support	11 (6.5)

#### Type of Intervention

In platforms designed for treatment purposes, the incorporation of ≥2 intervention types was common (20/169, 11.8%). The most common types of intervention were cognitive behavioral therapy (CBT; 50/169, 29.6%), problem-solving therapy (11/169, 6.5%), and psychoeducation (9/169, 5.3%). An average of 8 (SD 5.04) modules was offered by 41.4% (70/169) of platforms that provided module-based sessions. The common frequency of intervention was weekly (32/169, 18.9%), followed by daily (8/169, 4.7%) on the (42/169, 24.9%) platforms that reported this characteristic. Of note, some (5/169, 3%) platforms incorporated some form of in-app human interaction, where the interaction personnel included medical physicians (1/5, 20%; Ascend), psychologists (1/5, 20%; HelpID), therapists (1/5, 20%; SmartCAT), trained health care professionals (1/5, 20%; PratenOnline), Master’s degree–level students in clinical psychology (2/5, 40%; Happy@Work and Ascend), and occupational social workers (1/5, 20%; Happy@Work).

#### Type of Technology

Various types of technology have been deployed to fulfill the purposes of the mHealth platforms. Some (22/169, 13%) platforms used ≥1type of technology, with top choices including web-based interventions (90/169, 53.3%) and mobile apps (57/169, 33.7%; Table 4).

**Table 4:** Types of technology used by the platforms made for research purposes (N=169).

Technology type	Platforms, n (%)
Web-based platforms	90 (53.3)
Mobile apps	57 (33.7)
Text messaging	12 (7.1)
Others	6 (3.6)
Not specified	25 (14.8)

#### Additional mHealth Characteristics

Of the 169 platforms, 9 (5.3%) were available in ≥2 languages (Table 2). Of these, the most common languages besides English were German (4/9, 44%) and Spanish (4/9, 44%).

### App Store Search

#### Selection

**Figure 2:**
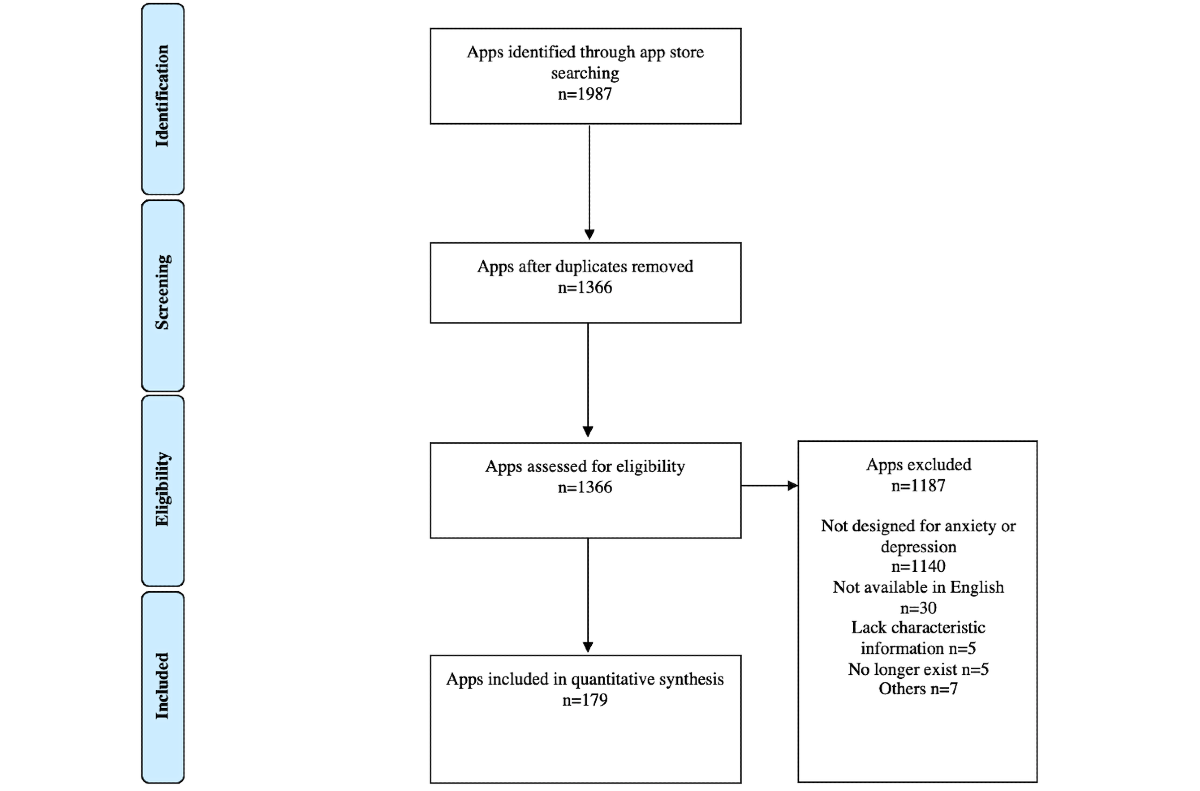
PRISMA (Preferred Reporting Item for Systematic Reviews and Meta-Analyses) flow diagram of search strategy and the finalized number of platforms included in the app store search for this review.

The search terms yielded 179 mHealth platforms from the selection of apps in the Singapore region’s Google Play Store and Apple App Store (Figure 2; Multimedia Appendix 3).

#### Commercial Availability

More apps were available in the Google Play Store (140/179, 78.2%) than in the Apple App Store (61/179, 34.1%; Table 5). Approximately 12.3% (22/179) apps were available in both app stores.

**Table 5:** Summary of characteristics of commercially available mHealth platforms (N=179).

Characteristics			Google Play Store (n=140), n (%)		Apple App Store (n=61), n (%)		Both^a^ (n=22), n (%)		All, (n=179), n (%)
**Targeted condition**									
	Anxiety	58 (41.4)		27 (44.3)		12 (54.6)		73 (40.8)	
	Depression	39 (27.9)		13 (21.3)		2 (9.1)		50 (27.9)	
	Both	43 (30.7)		21 (34.4)		8 (36.4)		56 (31.3)	
**Purposes**									
	1	77 (55)		32 (52.5)		7 (31.8)		102 (57)	
	2	45 (32.1)		16 (26.2)		7 (31.8)		54 (30.2)	
	≥3	18 (12.9)		13 (21.3)		8 (36.4)		23 (12.9)	
**Frequency of intervention**									
	Daily	4 (2.8)		3 (4.9)		0 (0)		7 (3.9)	
	Weekly	1 (0.7)		2 (3.3)		1 (4.6)		2 (1.1)	
	Not specified	135 (96.4)		56 (91.8)		21 (95.5)		170 (95)	
**Number of downloads**									
	<5000	67 (47.9)		4 (6.6)		4 (18.2)		67 (37.4)	
	5000-10,000	12 (8.6)		1 (1.6)		1 (4.6)		12 (6.7)	
	10,000-50,000	26 (18.6)		3 (4.9)		3 (13.6)		26 (14.5)	
	50,000-100,000	8 (5.7)		0 (0)		0 (0)		8 (4.5)	
	100,000-500,000	14 (10)		5 (8.2)		5 (22.7)		14 (7.8)	
	500,000-1,000,000	3 (2.2)		1 (1.6)		1 (4.6)		3 (1.7)	
	>1,000,000	6 (4.3)		4 (6.6)		4 (18.2)		6 (3.4)	
	Not available	4 (2.9)		43 (70.5)		4 (18.2)		43 (24)	
**Ratings^b^**									
	1 star	1 (0.7)		1 (1.6)		0 (0)		2 (1.1)	
	2 stars	0 (0)		1 (1.6)		0 (0)		1 (0.6)	
	3 stars	7 (5)		4 (6.6)		0 (0)		10 (5.6)	
	4 stars	58 (41.4)		21 (34.4)		10 (45.6)		70 (39.1)	
	5 stars	43 (30.7)		33 (54.1)		12 (54.6)		64 (35.8)	
	Not specified	31 (22.1)		1 (1.6)		0 (0)		32 (17.9)	
**Cost**									
	Free	85 (60.7)		19 (31.2)		4 (18.2)		100 (55.9)	
	Free and paid versions available	39 (27.9)		30 (49.2)		13 (59.1)		56 (31.3)	
	Payment required	16 (11.4)		12 (19.7)		5 (22.7)		23 (12.8)	

^a^Both refer to platforms found in both Google Play Store and the Apple App Store.

^b^Ratings were rounded to the nearest integer.

#### Targeted Condition

The mHealth apps identified in both app stores were designed predominantly for anxiety (73/179, 40.8%). Approximately 30.7% (43/140) of the apps in the Google Play Store and 34% (21/61) of the apps in the Apple App Store claimed to address both anxiety and depression (Table 5).

#### Targeted Group

All platforms provided suitable age groups. One of the platforms, the Geriatric Depression Scale 2.0, detailed a specified target group, that is, older adults.

#### Purpose

Most apps found in both stores offered 1 purpose (Android devices: 77/179, 43%; iOS devices: 32/179, 17.9%; Table 5). Among the identified purposes available through the apps, treatment was the most common purpose in both app stores, with 72.1% (129/179) of the apps including it (Table 6).

**Table 6:** The primary purpose mobile health platforms for anxiety or depression identified from the Google Play Store and Apple App Store (N=179).

Purpose	Description	Examples	Apps, n (%)
Education	Provide information and communication of self-monitoring procedures Dissemination of information by health care professionals	Text-based information, video materials, and SMS text messaging	50 (27.9)
Diagnostic	Interpret symptoms Identify possible mental health conditions	Self-administered questionnaires and psychological tests	34 (19)
Treatment	Offer treatment tips, recommendations, and advice Provide some form of intervention	SMS text messaging, real-time videoconferencing, and text-based treatment suggestions	129 (72.1)
Monitoring	Tracking of symptoms Prompts and reminders for tasks and appointments	In-app notifications, self-administered questionnaires, and journaling	26 (14.5)
Support	Provide access to some form of support Features option to call a friend, a family member, health care professionals, or a nearby help center directly from the platform	Forums, chatbots, and SMS text messages	34 (19)
Prevention	Claim to prepare for and prevent anxiety-related or depressive episodes Offer modules or activities that serve as preventive measures	Text-based information, audio, and activities	8 (4.5)

#### Type of Intervention

A total of 22 types of interventions were identified from the 72.1% (129/179) of apps that offered some form of treatment. Of these, referral to care or counseling (36/129, 27.9%), problem solving (27/129, 20.9%), and CBT (22/129, 17.1%) were the most common types of interventions available. Apart from encouraging to seek or directly offering specialist support, some apps provided support from the community, for example, access to forums with like-minded users (16/179, 8.9%). A subset of the apps that offered referral (14/179, 7.8%), counseling (4/179, 2.2%), or forum services (2/179, 1.1%) was subscription-based or required payment to gain access.

#### Type of Technology

Of the 179 platforms, all the platforms were available for mobile devices, except for 1 (0.6%; At Ease Anxiety and Worry Relief), which was accessible from both iOS and macOS devices. Of these 179 platforms, 140 (78.2%) were available on the Android operating system, 61 (34.1%) were available on iOS, and 22 (12.3%) were available on both operating systems. Other notable forms of technology that complemented or were incorporated in the platforms included wearable devices, such as headsets or smartwatches (1/179, 0.6%), and artificial intelligence–backed counseling sessions (3/179, 1.7%).

#### App Store Categorization

The app categorization and classification differed between the 2 stores. In Google Play Store, mHealth apps were categorized into 14 categories (Health and Fitness, Medical, Lifestyle, Books and Reference, Education, Trivia, Casual, Simulation, Adventure, Puzzle, Music and Audio, Social, Personalization, and Productivity), whereas Apple App Store mHealth apps were categorized into 5 categories (Health and Fitness, Medical, Lifestyle, Games, and Social Networking). The Health and Fitness category was the most common classification of mHealth platforms available on both the Google Play Store (81/179, 45.3%) and Apple App Store (39/179, 21.8%). The Medical category was the second most common classification available on both the Google Play Store (27/140, 19.3%) and Apple App Store (18/61, 29.5%).

#### Additional mHealth Characteristics

Of the 179 mHealth platforms, 100 (55.9%) were made freely available, with 56 (31.3%) providing both free and paid versions and 23 (12.8%) offering only paid versions. The costs of platforms that required payments ranged from SGD 1.21 (US $0.90) per installation to SGD 399.99 (US $296.20) for lifetime use.

### Clinical Evidence and Regulatory Information for Literature and App Store mHealth Platforms

RCTs are clinical evidence and a prerequisite for regulatory approval. However, not all platforms had RCTs. Additional regulatory information—RWE and regulatory approval—was searched. In the secondary search mentioned in Table 1, a subset of apps had a credible source that offered some form of empirical evidence. In the app store searches, 3.4% (6/179) of the apps included scientific evidence claims, whereas 2.8% (5/179) of the apps were affiliated with health care experts and reputable institutions.

None of the apps in both literature and app store searches were reported as medical devices. In the literature search, 44.4% (75/169) and 45% (76/169) of articles presented evidence from RCTs and RCT protocols (Table 7), respectively, whereas the remaining 10.7% (18/169) did not provide evidence from RCTs. Further analysis of the clinical and regulatory evidence—through the search for additional RCTs, RWE, and regulatory approvals—demonstrated that 41.4% (70/169) of platforms presented evidence from RCTs, 0.6% (1/169; Wysa) presented preliminary RWE, 1.2% (2/169; myStrength and Moodgym) presented evidence from RCTs and RWE, and 0.6% (1/169; Deprexis) presented evidence from RCTs and RWE and had obtained regulatory approval.

From the app store search, a subset of apps was pinpointed to be based on substantial validation studies to assess effectiveness. In particular, RWE can serve as a key gateway and vitally important indicator for supporting health care decisions, as well as impact and value toward patient care and the broader health care ecosystem. For example, 1.1% (2/179) of the apps provided evidence from each of the following sources: RCTs (Sanvello and Moodmission); RWE (Talkspace and Wysa); RCTs, RWE, and regulatory approval when applicable (Flow and Sooma).

**Table 7:** Availability of clinical-grade evidence and regulatory oversight in mobile health platforms for anxiety or depression identified from the literature and app store searches.

Evidence	Literature search (N=169), n (%)	App stores search (N=179), n (%)
RCTs^a^ protocol	75 (44.4)	—^b^
RCTs	76 (45)	2 (1.1)
RWE^c^	—^b^	2 (1.1)
RCT+RWE	—^b^	0 (0)
RCT+RWE + regulatory approval	—^b^	2 (1.1)
None	18 (10.7)	173 (96.7)

^a^RCT: randomized controlled trial.

^b^No reported clinical evidence or additional regulatory information found.

^c^RWE: real-world evidence.

## Discussion

### Principal Findings

This review provides an in-depth analysis of the current mHealth platforms developed for anxiety or depression available for research and commercial purposes. Through a novel combined approach—a 2-pronged systematic search across the research literature and marketplace—we identified 169 and 179 mHealth platforms, respectively. Most platforms developed for research purposes were designed for depression (116/169, 68.6%), whereas the platforms made available in the app stores were mainly developed for anxiety (Android: 58/179, 32.4%; iOS: 27/179, 15.1%). We identified that mHealth characteristics such as platform name, aspects of mental health addressed, purpose, type of intervention, type of technology, and level of evidence varied largely across these platforms. In addition, we highlighted a significant lack of adoption of the regulatory framework and standards governing these mHealth platforms. An overview of the existing platforms for anxiety and depression available for research, commercial use, or both, as reported in this review, allowed us to identify gaps in the literature and marketplace and subsequently provide guidance for recommendations for future frameworks of mHealth development and regulations.

### Targeted Group, Purpose, and Intervention Type of Available mHealth Platforms for Anxiety and Depression

Among the abundance of mHealth options currently available for anxiety or depression, relatively few have been developed for youths and emerging adults [167,168]. This is surprising, as these age groups are among the most prevalent users of mobile technologies as well as the groups that are most in need of mental health interventions. In particular, typical engagement with standard care for mental health has low appeal to young people and can be antagonistic to their developmental needs [168]. The low motivation inherent in emotional disorders; the need for agency, anonymity, and privacy; the greater sensitivity to stigma and discrimination associated with mental health; and the development of self-monitoring among young people can be addressed by mHealth approaches that provide personalized and timely treatment [169,170]. Future developers of mHealth platforms for anxiety or depression should consider developing them with this targeted group in mind.

The main purpose of most of the mHealth platforms for anxiety or depression identified in this review focused on treatment. The treatment used features such as module-based sessions and the teaching of coping and management strategies, which were based on the principles and guidelines of CBT, problem-solving therapy, and psychoeducation. Previous research has revealed 3 primary areas for enhancing the effectiveness of the treatment function of mHealth [167]. First, mHealth needs to be complemented by human interactions, especially with a medical physician or health care worker, to increase compliance, adherence, dropout [2,171,172], and efficacy in treatment [173-175]. However, findings from this review highlighted that few mHealth platforms included human interactions. To be effective, the digital framework for human interaction needs to consider individuals’ desire for anonymity and privacy. Second, initial engagement and sustained use of mHealth platforms for young people require some form of social support from peers, schools, or mental health professionals. Although the role of social support and the associated outcomes are similar to that found with the standard of care, the distinct feature of digital social support that mHealth platforms provide is anonymity and accessibility. The opportunity to share similar lived experiences of anxiety or depression with one’s peers and being accountable to an authority (eg, health care professional) for the purpose of monitoring or assessment of their symptoms has the potential to increase their engagement and adherence to the mHealth platforms. Notwithstanding the support function of mHealth platforms, few platforms included this in their design feature. Finally, an mHealth platform needs to be easy to navigate, relatable, engaging, and esthetically appealing in providing education for mental health users—another common function of mHealth platforms. Previous studies have found that the boring and repetitive nature of modules and lessons presents a barrier to greater engagement with the education feature of an mHealth platform [10].

### Type of Technology

mHealth can be deployed via one or a combination of mobile technologies. An increasing trend, which is also observed in health care, is to reach the individuals by communication technologies that are already in use for the seamless incorporation of the intervention into their daily life. Our literature search reported web-based platforms (90/169, 53.3%), mobile apps (57/169, 33.7%), and messaging (12/169, 7.1%) as the most common modalities deployed, which is consistent with findings in other studies [176]. In our literature search, 13% (22/169) of the platforms used ≥1 type of technology.

The app stores search identified discrepancies in the number of apps available in each app store. The higher number of apps in the Google Play Store (140/179, 78.2%) than in the Apple App Store (61/179, 34.1%) can be attributed to a preference for the Android operating system over iOS in Asia (82.7% vs 16.5%) and Singapore (65.2% vs 33.9%), as recorded in June 2020 [177]. Although the infrequent download count data from iOS disables the comparison in this category in our review, the literature suggests that in the United States, the iOS preference is reversed (41.7% vs 58.2% for Android and iOS, respectively) [177], and iOS apps show a higher number of downloads of behavioral health mobile apps [178]. Interestingly, a recent study collecting location data has shown that the platform type had a measurable effect on the retention of passive data collection because of the increased battery consumption of the app for the iOS system. In addition, the operating systems differ in their battery-saving modes and memory space–saving strategies, for example, app offloading to a cloud, which may have further implications. In the same study, no differences were detected in active data collection retention, suggesting that the operating system had no effect on active user interactions with the mHealth platform [179].

The deployment technology type and its specific technical considerations (eg, operating system) can *make or break* an mHealth platform. A digital intervention design should carefully consider the context of how each technology is already being used, as well as its technical and behavioral limitations. An mHealth platform can be deployed either via one technology type only or in a hybrid model to reap the benefits of each technology type to maximize usability with sustained functionality toward the highest efficiency of the intervention.

### Commercial Versus Academic Motivation Considerations

Of note, only 4.7% (8/169) of the mobile apps identified in the literature search were available in app stores. This can be potentially related to the geographical constraints of the app store search compared with the unconstrained geography of the literature search. In addition, the literature searches covered articles from inception to 2020, whereas the app store search included only the apps present in the marketplace in June 2020. It is not unusual that after the gathering of evidence, the apps become unavailable in the marketplace [178,180,181]. The lack of commercial translation of research published in literature can be attributed to multiple factors: funding timelines (eg, relatively short grant duration and high staff rotation may not enable long term app management) and downstream research objectives (basic or fundamental research, tool development, and proof-of-concept to support potential future commercial and large-scale deployment) [182].

From the perspective of the marketplace, 1.7% (3/179) of apps in the app store search were described in a peer-reviewed article identified in our search. This can be potentially attributed to a business strategy that favors *quick-in* and *quick-out* rather than laborious and costly app evaluation without prevailing guidelines or incentives—monetary, regulatory, or otherwise—in doing so. Although consumers make the initial decision of which app to download based on easy-to-judge attributes such as graphics, price, and rating [178,183], it is the credibility and trustworthiness that drive engagement with mental health apps [184-186]. With this realization, it is important for commercial app developers to generate scientific evidence that validates their apps to substantiate the scientific descriptions, which will collectively enhance the app’s appeal. In a study of the app descriptions of the top-ranked apps, it was previously found that apps use scientific jargon and are not supported by principles backed by peer review and validation studies, or they do not align with established findings in the scientific literature [187]. Instead of objectively informing about the evidence, the function of the app description is to appeal to consumers and drive the number of app downloads, independent of the platform’s objective quality [187]. Accordingly, app user ratings do not indicate clinical utility or quality [188,189].

### Regulatory Information: Scientific Evidence and Regulatory Status of mHealth Platforms for Research and Commercial Use

Clinical evidence, in the form of RCTs, reported in the publications found in the literature search (151/169, 89.4%) vastly exceeded that for apps found in the app store (2/179, 1.1%). Regulatory approvals (0.6% vs 1.1%) and RWE (0.6% vs 1.1%) were more balanced between platforms from the literature and app stores. The relatively high frequency of RCTs but low numbers in RWE and regulatory approvals for platforms featured in the literature search seem to indicate a pre-eminently investigative intent of those platforms. In addition, 33.7% (57/169) of the platforms were available as mobile apps, further hinting at treatment commercialization as a secondary possibility. Maintenance and update of the website platforms were, in some cases, not frequent, indicating ad hoc use.

A large proportion of the mHealth platforms from the app store were unregulated, clinically untested, and unevaluated for real-world effectiveness (173/179, 96.7%), indicating a potential need for business models that support investment in robust clinical or real-world validation. Importantly, although 1.7% (3/179) of the apps found in the app store search in this review could claim to have scientific evidence or regulatory approval, 15.1% (27/179) and 10.1% (18/179) of the mHealth platforms for anxiety or depression extracted from the Google Play Store and Apple App Store, respectively, were included in the Medical category. Under this category (excluding telehealth), 8.4% (15/179) of the platforms affirmed that health professionals were involved in the design of the apps. From the app stores, 8.9% (16/179) of the platforms included a disclaimer notifying that the app was not intended to be a replacement for treatment or any sort of medical intervention. Another 12.3% (22/179) of the apps mentioned information sources used to design the app, such as validated tests (eg, Patient Health Questionnaire-9 for the assessment of depression and Generalized Anxiety Disorder-7 for the assessment of anxiety), international organization guidelines (eg, World Health Organization), validated therapies (eg, CBT), or peer-reviewed articles. These numbers reflect the need to develop more actionable and meaningful app store categorizations for medical apps. In addition, increased app store categorization oversight and strategies to enable or incentivize owners to implement changes, as well as clearer accountability guidelines pertaining to store content quality, may be needed. For example, oversight could be extended to app store owners to assist in enhancing specificity regarding the medical classification of apps. In addition, greater user education in the selection of apps may be a downstream consideration for health authorities. Consumers may need to be able to discern real-value apps by analyzing their claims, the involvement of health professionals, regulatory status, and scientific evidence. Apps that have not been supported by established validation guidelines adversely affect the field and users in multiple ways. For example, they may add a burden to already strained health care systems. In addition, they may create opportunity costs when the technological promise is not realized, resulting in mHealth fatigue of the clinicians, patients, and consumers, thus delaying the implementation of evidence-based, efficient mHealth platforms.

Overall, a need for enhanced regulation, especially in the Medical category of the stores, indicates that authorities may be able to positively affect the mHealth landscape toward requirements that include threshold validation and other applicable compliance guidelines. Several attempts have been made in that direction from various government organizations and professional societies. For example, the Institute of Electrical and Electronics Engineers—an engineering professional society—developed the Institute of Electrical and Electronics Engineers 1752 standards in 2017, which define specifications for standardized representations for mHealth data and metadata for cardiovascular, respiratory, and metabolic measures [190]. Health Level Seven International—an international mHealth professional workgroup—released the Consumer Mobile Health Application Functional Framework in 2018 with the primary goal of providing a standard whereby a mobile app’s characteristics can be assessed [191]. In 2020, the American Psychiatric Association—a professional organization of psychiatrists—developed the American Psychiatric Association App Advisor, an initiative to identify an app evaluation model that was specific to mental health to be used by clinicians and their patients [192]. Most recently, in February 2021, a joint unit formed by the National Health Service England and the Department of Health and Social Care*—*released the Digital Technology Assessment Criteria with the aim to set the expectation of digital health technologies for potential developers [193]. In addition, other digital health and medicine professional societies, such as the Digital Medicine Society and Digital Therapeutics Alliance, also have initiatives to develop frameworks, standards, and resources for the different stakeholders of digital health interventions [194,195]. Furthermore, the International Organization for Standardization Technical Specification, in collaboration with the European Committee for Standardization, is currently developing a health app quality label *ISO/TS 82304-2 Health software–Part 2: Health and wellness apps–Quality and reliability* [196,197]. Although the continued development of these regulatory frameworks is critical, to have a real-world impact on the quality of mHealth platforms and apps, they must also be widely adopted by developers and requested by regulators, marketplace owners, and target populations alike. This will ultimately result in the development of high-quality mHealth platforms with stringent safety and security measures required in health care.

### Methodological Limitations and Further Considerations

Although we took a novel approach of combining both a systematic literature search and app store search, some mHealth platforms may have been excluded. For example, although we had no geographical filters in our literature search, our commercially available app search was based on regional availability in Singapore’s Google Play Store and Apple App Store. Although this may be a region-specific search, this review also provides new insight into the field as it serves as an early resource detailing available mHealth apps and platforms in South-East Asia. As Singapore is a globally recognized regional information communications technology hub with a well-established and efficient health care innovation ecosystem, this review provides the basis for a review of commercially available mHealth platforms available in surrounding countries and worldwide, alongside actionable recommendations. In addition, we restricted our inclusion criteria to mHealth platforms in English. It is possible that some apps may be published exclusively in other countries’ stores (eg, United States, United Kingdom, or Canadian stores) and in other languages that were ultimately excluded from our search and this review. Furthermore, in the academic setting, the goal of the research team may be to assess the efficacy of the mHealth intervention they developed. After proven efficacious, commercialization may be the next step in the pipeline, in which case a name may be given to the platform. As such, this review does not include nameless mHealth platforms that were developed solely for research purposes. This decision was made so that we could identify the app crossover between the 2 searches. In addition, we used exclusion criteria such as mHealth platforms developed for specific target populations (eg, comorbidities), secondary mental health problems related to a physical condition (eg, pregnancy), and platforms targeting health behaviors (eg, anxiety for smoking cessation) and severe mental illnesses. These criteria narrowed down the included platforms considerably. Overall, the possibility of mHealth platform exclusion may have introduced bias into our analysis. However, by including a comprehensive search of both the literature and multiple app stores, we aimed to minimize the possibility of mHealth platform exclusion in this review. Finally, as previously mentioned, we carefully selected search terms that could be used in both the literature and app store searches to ensure that these searches could be conducted as homogeneously as possible despite the different methodological requirements for each. As a result, some common key terms (eg, treatment and prevention) and phrases for the clinical conditions (eg, mood, sadness, affective disorder, phobias, and CBT) that would be included in a traditional literature database search were excluded. Therefore, it is plausible that some mHealth platforms described in the literature may have been excluded from this review. However, this review aimed to provide an overview of current mHealth platforms developed specifically for general nonspecific anxiety or depression; thus, we do not expect many potentially excluded platforms from this review. If more specific anxiety and depression conditions such as mood disorders or phobias are of interest, the keywords should be developed accordingly.

Furthermore, this review does not include a quality assessment of the mHealth platforms. We intended to use the Mobile App Rating Scale [198], as indicated in our PROSPERO registration (CRD42020193956). However, it was beyond the scope of this project to download all the included mHealth platforms, many of which required payment for installation or were not accessible (ie, from the literature). Therefore, we used the information detailed in the descriptions provided by the app stores or in the literature. As a result, some data were not available, and we could not avail of the Mobile App Rating Scale for the intended purpose. We attempted to use an alternative mHealth quality assessment tool; however, we did not find one that would be suitable for these purposes. Nouri et al [199] has previously identified substantial heterogeneity in the assessment criteria for mHealth apps in different studies. As valid and reliable frameworks to assess the quality of mHealth platforms are developed, future reviews, such as ours, should incorporate them.

### Future Directions: Digital Therapeutics as the Next Step for mHealth Solutions for Anxiety and Depression

It is estimated that 264 million people have depression and 284 million people have anxiety worldwide [200]. The COVID-19 pandemic may exacerbate these numbers [201]. Although effective treatments exist, many health systems worldwide are underresourced and struggling to respond to the burden of anxiety, depression, and other mental health disorders effectively and efficiently [201]. As a result, mHealth platforms continue to gain popularity and are emerging as a feasible option for improving the quality of and overcoming access barriers to mental health support. However, the aforementioned concerns of lack of regulation and low levels of evidence are limiting factors for the current mHealth platforms developed for anxiety and depression to become successful solutions. A leap forward would involve venturing into digital therapeutics (DTx), which is widely regarded as the next step for mHealth. DTx offers effective, safe, highly personalized, objective, and cost-saving health care where barriers between information silos are overcome [202], all of which are ideal for addressing mental health conditions such as anxiety and depression. DTx require clinical and regulatory oversight and demonstrated RWE and are further differentiated by the delivery of a clinical-grade intervention (beyond tracking) to treat or manage a disease [198]. In other words, DTx interfere with the patient to modify their state, often through behavioral alteration. They can operate as a standalone treatment (eg, treating a mental disorder through gamification) or adjunct to other therapies to enhance them (eg, improving drug adherence). With the expectation to grow dramatically over the next few years, DTx can potentially affect users, organizations, and the health care industry in different ways: new treatment options, improved care pathways, incorporation of new standards of care, improved patient and population health outcomes, similar coverage to existing therapies, international product quality standards, and acceptance as an independent class of medicine, among others [203]. DTx is a favorable next step in mHealth solutions for anxiety and depression.

### Conclusions

This study analyzed the characteristics of mHealth platforms for anxiety or depression that were available for research, commercial use, or both. A systematic search of the current literature and 2 popular app stores identified 169 and 179 mHealth platforms, respectively. mHealth platforms varied in their targeted condition, targeted group, purpose, intervention type, and technology type. In addition, most platforms from popular app stores lacked clinical and RWE, and a small number of platforms present in the literature were available commercially. Future efforts should focus on further accessing the quality—utility, safety, and effectiveness—of the existing platforms and ensuring the adoption by developers, from both commercial and academic sectors alike, of a reporting guideline for their platform description, as well as a regulatory framework to facilitate the development, validation, and deployment of mHealth platforms for anxiety or depression.

## Appendix

Multimedia Appendix 1 Table of search strings used across the 4 databases and their respective numbers of outcomes.

Multimedia Appendix 2 Table of brief mobile health platform characteristics from the literature search.

Multimedia Appendix 3 Table of brief mobile health platform characteristics from the app store search.

## Abbreviations

CBT: cognitive behavioral therapy
DTx: digital therapeutics
mHealth: mobile health
PRISMA: Preferred Reporting Items for Systematic Reviews and Meta-Analyses
PROSPERO: International Prospective Register of Systematic Reviews
RCT: randomized controlled trial
RWE: real-world evidence
