# Supplementary material for: Characteristics of Mobile Health Platforms for Depression and Anxiety: Content Analysis Through a Systematic Review of the Literature and Systematic Search of Two App Stores
Source: J Med Internet Res. 2022 Feb 4;24(2):e27388. doi: 10.2196/27388 (PMC8857696; doi:10.2196/27388)
Supplement: Multimedia Appendix 1 [file jmir_v24i2e27388_app1.docx]

**Appendix**

**Appendix 1: Table of search strings used across four databases and their respective number of outcomes.**

| **Database** | **Search string** | **Number of outcomes** |
| --- | --- | --- |
| Pubmed | ((("mental health"[All Fields] OR "mindfulness"[All Fields]) OR "anxiety"[All Fields]) OR "depression"[All Fields]) AND ((((((("mHealth"[Title/Abstract] OR "mobile health"[Title/Abstract]) OR "mobile device"[Title/Abstract]) OR "mobile application"[Title/Abstract]) OR "digital therapeutics"[Title/Abstract]) OR "digital therapeutic"[Title/Abstract]) OR "digital intervention"[Title/Abstract]) OR "ehealth"[Title/Abstract] OR “smartphone”[Title/Abstract] OR “mobile phone”[Title/Abstract] OR “text message”[Title/Abstract] OR “web based”[Title/Abstract] OR “web application”[Title/Application) | 5289 |
| EMBASE | ('mental health' OR mindfulness OR anxiety OR depression) AND (mhealth:ab,ti OR 'mobile health':ab,ti OR 'mobile device':ab,ti OR 'mobile application':ab,ti OR 'digital therapeutics':ab,ti OR 'digital therapeutic':ab,ti OR 'digital intervention':ab,ti OR 'ehealth':ab,ti OR 'smartphone':ab,ti OR 'mobile phone':ab,ti OR 'text message':ab,ti OR 'web based':ab,ti OR 'web application':ab,ti) | 6528 |
| CINAHL | MW ( 'mental health' OR mindfulness OR anxiety OR depression ) AND TI ( mhealth OR 'mobile health' OR 'mobile device' OR 'mobile application' OR 'digital therapeutics' OR 'digital therapeutic' OR 'digital intervention' OR 'ehealth' OR 'smartphone' OR 'mobile phone' OR 'text message' OR 'web based' OR 'web application' ) | 700 |
| PsychInfo | ('mental health' or mindfulness or anxiety or depression).mh. and (mhealth or 'mobile health' or 'mobile device' or 'mobile application' or 'digital therapeutics' or 'digital therapeutic' or 'digital intervention' or 'ehealth' or 'smartphone' or 'mobile phone' or 'text message' or 'web based' or 'web application').ti. | 165 |
| **Total** | | **12682** |
