# Supplementary material for: Characteristics of Mobile Health Platforms for Depression and Anxiety: Content Analysis Through a Systematic Review of the Literature and Systematic Search of Two App Stores
Source: J Med Internet Res. 2022 Feb 4;24(2):e27388. doi: 10.2196/27388 (PMC8857696; doi:10.2196/27388)
Supplement: Multimedia Appendix 2 [file jmir_v24i2e27388_app2.docx]

**Appendix**

**Appendix 2: Table of brief mHealth platform characteristics from the literature search**

| **Reference** | **Name of mHealth platform** | **Targeted condition** | **App store availability** | **Purpose** | **Type of technology** | **Type of intervention** |
| --- | --- | --- | --- | --- | --- | --- |
| [59] | SPARX | Depression | - | Treatment | Mobile App | CBT, Gamification |
| [60] | GET.ON-Mood Enhancer-WL | Depression | - | Education, Treatment | Web-based | Psychoeducation, CBT, PST |
| [61] | myStrength | Depression | - | Treatment | Web-based | computerised CBT |
| [62] | Sonreír es Divertido | Depression | - | Education, treatment | Web-based | Interactive media |
| [63] | iDOVE | Depression | - | Prevention | Text messaging | - |
| [64] | RecoveryRoad | Depression | - | Education, Monitoring, Treatment | - | - |
| [65] | Youth StepCare | Anxiety, Depression | - | Diagnostic | Web-based | - |
| [66] | CATCH-IT | Depression | - | Prevention | Web-based | CBT, BA, Interpersonal therapy |
| [67] | MoodMission | Anxiety, Depression | Apple App Store, Google Play Store | Treatment | Mobile App | CBT |
| [68] | iHOPE | Depression | Apple App Store, Google Play Store | Monitoring | Mobile App | EMA |
| [69] | MARIGOLD | Depression | - | Treatment | Web-based | Psychoeducation |
| [70] | Mindful Mood Balance | Depression | - | Education, treatment | Web-based | Mindfulness-based CT (MBCT) |
| [71] | MoodMission | Anxiety, Depression | - | Treatment | Mobile App | - |
|  | Pacifica | Anxiety | - | Monitoring, Treatment | Mobile App | - |
|  | Mindshift | Anxiety | - | Monitoring, Treatment | Mobile App | - |
|  | What's Up? | Depression | - | Education, Treatment | Mobile App | - |
| [72] | Lantern | Anxiety | - | Education, Treatment | Mobile app, Text messaging, Web-based | CBT, Motivational behavioral coaching |
| [73] | Living to the Full | Anxiety | - | Treatment | - | ACT |
| [74] | MoodMonitor | Depression | - | Monitoring | Mobile App | EMA |
| [75] | Thought Challenger | Depression | - | Treatment | Mobile App | BA |
|  | Boost Me | Depression | - | Treatment | Mobile App | CT |
| [76] | TODAY! | Anxiety, Depression | - | Education, Monitoring, Treatment, Support | Mobile App | Transdiagnostic CBT |
| [77] | K-CESD-R Mobile | Depression | Google Play Store | Diagnostic | Mobile App | - |
| [78] | REACH | Anxiety | - | Prevention, Treatment | Mobile App | Exposure-based CBT |
| [79] | Improvehealth.eu | Depression | - | Treatment | Web-based, Text messaging | - |
| [80] | Kokoro-no-skill-up-training | Depression | - | Education, treatment | Web-based | CBT |
| [81] | Dorehye Amozeshie Dokhtaran (DAD) internet intervention program | Depression | - | Treatment | Web-based | - |
| [82] | Deprexis | Depression | - | Education, Treatment | Web-based | CBT |
| [83] | eSMART-MH | Depression | - | Treatment | - | Gamification |
| [84] | The Journal | Depression | - | Treatment | Web-based | BA, Problem-solving |
| [85] | Happy@Work | Depression | - | Treatment | Web-based, Text messaging | - |
| [86] | MoodGYM | Depression |  | Treatment | Web-based | CBT, Gamification |
| [87] | Ascend | Depression | - | Treatment, Support | Mobile App and Text messaging | CBT |
| [88] | Icare Prevent | Anxiety, Depression | - | Education, Treatment | Web-based | CBT |
| [89] | Happy@Work | Depression | - | Treatment | Web-based, Text messaging | PST. CT |
| [90] | Deprexis | Depression | - | Treatment | Web-based | CBT |
| [91] | SmartCAT | Anxiety | - | Treatment, Support | Mobile App, Web-based | CBT and EMI |
| [92] | Partners in Parenting (PiP) program | Anxiety, Depression | - | Prevention | Web-based | - |
| [93] | Partners in Parenting (PiP) Level 4 | Anxiety, Depression | - | Prevention | Web-based | - |
| [94] | iCBT program | Anxiety, Depression | - | Education, Treatment | Web-based | CBT |
|  | Mindfulness-Enhanced iCBT program (MEiCBT) | Anxiety, Depression | - | Education, Treatment | Web-based | CBT, BA and CT |
|  | The Mindfulness Training Program (iMT) | Anxiety, Depression | - | Education, Treatment | Web-based | - |
| [95] | MoodGYM | Depression | - | Treatment | Web-based | CBT |
|  | BluePages | Depression | - | Education | - | - |
| [96] | BiP Anxiety | Anxiety | - | Treatment | Web-based, Text messaging | CBT |
| [97] | Deprexis | Depression | - | Education, treatment | Web-based | CBT |
| [98] | Pacifica Premium | Anxiety, Depression | - | Monitoring, Treatment | Mobile App | - |
| [99] | Mindful Mood Balance | Depression | - | Education, Treatment | Web-based | Mindfulness-based CT |
| [100] | Smartphone and OnLineUsage-based eValuation for Depression (SOLVD) | Depression | - | Monitoring | Mobile App | - |
| [101] | DepWatch | Depression | - | Monitoring | - | - |
|  | LifeRhythm | Depression | - | Monitoring | - | - |
| [102] | Woebot | Anxiety, Depression | - | Treatment, Support | Web-based | CBT, gamification |
| [103] | Happy@Work | Anxiety, Depression | - | Treatment | Web-based | PST, CT |
| [104] | Mindfulness Virtual Community (MVC) | Anxiety, Depression | - | Treatment | Web-based | - |
| [105] | Get Happy Program | Depression | - | Treatment | Mobile App | CBT |
| [106] | Kokoro-App | Depression | - | Treatment | Mobile App | CBT, BA, Cognitive restructuring |
| [107] | SmartCAT 2.0 | Anxiety | - | Treatment | Mobile App, Web-based, Text messaging | CBT, Gamification |
| [108] | Web-Based Depression and Anxiety Test (WB-DAT) | Anxiety, Depression | - | Diagnostic | Web-based | - |
| [109] | Act and Feel' treatment | Depression | - | Education, Monitoring | Web-based | BA |
| [110] | Help for Depression (HDep) | Depression | - | Education, Monitoring, Support | Web-based | CBT, Psychoeducation |
| [111] | Net Decision Support System (NetDSS) | Depression | - | Support | Web-based | - |
| [112) | Her ey Kontrol Altında (H KA) | Depression | - | Treatment | Mobile app, Web-based | PST |
| [113] | MoodGYM | Depression | - | Treatment | Web-based | CBT |
| [114] | iPST | Depression | - | Treatment | Mobile App | PST, Gamification |
|  | EVO | Depression | - | Treatment | Mobile App | CT |
| [115] | Mobile Affect Regulation Intervention with the Goal of Lowering Depression (MARIGOLD) | Depression | - | Education, Treatment | Web-based | - |
| [116] | Deprexis | Depression | - | Treatment | - | - |
| [117] | MoodGYM | Depression | - | Treatment | Web-based | CBT |
|  | BluePages | Depression | - | Education | Web-based | - |
| [118] | Moodivate | Depression | - | Education, Treatment | Mobile app | BA |
| [119] | Partners in Parenting (PiP) | Anxiety, Depression | - | Treatment | Web-based | - |
| [120] | Entourage | Anxiety | - | Treatment, Support | Web-based | - |
| [121] | Ascend | Anxiety, Depression | - | Treatment | Mobile App | - |
| [122] | MOOD | Depression | - | Education, treatment | Web-based | - |
| [123] | iDOVE | Depression | - | Treatment, Support | CBT, Motivational interviewing | Text-messaging |
| [124] | myCompass | Anxiety, Depression | - | Monitoring, Treatment | Web-based | - |
| [125] | E-Couch | Anxiety | - | Education, Treatment | Web-based | CBT |
| [126] | SmartCAT | Anxiety | - | Treatment, Support | Mobile App, Web-based | Gamification |
| [127] | myCompass | Anxiety, Depression | - | Monitoring, Treatment | Web-based | - |
| [128] | http://www.baejy.com/smiles | Depression | - | Monitoring, Support | Web-based | - |
| [129] | IntelliCare | Anxiety, Depression | - | Treatment | Mobile App | - |
| [130] | Partners in Parenting (PiP) | Anxiety, Depression | - | Prevention, Treatment | Web-based | Psychoeducation |
| [131] | Allesondercontrole | Anxiety, Depression | - | Treatment | Web-based | PST |
| [132] | HelpID | Depression | - | Treatment, Support | Web-based | CBT, Mindfulness training, Systemic counseling |
| [133] | MedLink | Depression | - | Monitoring, Education, Support | Mobile App | - |
| [134] | Coordinated Anxiety Learning and Management (CALM) | Anxiety | - | Support | Web-based | CBT |
| [135] | MedLink | Depression | - | Education, monitoring, support | - | - |
| [136] | BRAVE-ONLINE | Anxiety | - | Treatment | Web-based | CBT |
| [137] | Short-Term Depression Detection (STDD) | Depression | - | Diagnostic, Monitoring | Mobile App | - |
|  | Headgear | Depression | - | Diagnostic, Monitoring | - | - |
|  | Socialise | Depression | - | Diagnostic | - | - |
|  | Purple robot | Depression | - | Diagnostic, Monitoring | - | - |
|  | FINE | Depression | - | Diagnostic, Monitoring | - | - |
|  | Mobylize! | Depression | - | Diagnostic, Monitoring, Prevention | - | - |
|  | iHOPE | Depression | - | Monitoring | - | - |
|  | iBobbly | Depression | - | Prevention | - | - |
| [138] | ThisWayUp | Anxiety | - | Education, Support | Web-based | CBT |
| [139] | The Journal | Depression | - | Treatment, Support | Web-based | - |
| [140] | i-RFCBT | Depression | - | Treatment | Web-based, Text messaging | Rumination-focused CBT |
| [141] | Deprexis | Depression | - | Treatment | Web-based | - |
| [142] | Psychologist in a Pocket (PiaP) | Depression | - | Diagnostic, Monitoring | Mobile App | - |
| [143] | FINE | Depression | - | Support | Mobile App | - |
| [144] | IntelliCare | Anxiety, Depression | - | Treatment | Mobile App | - |
| [145] | Headgear | Depression | Apple App Store, Google Play Store | Treatment | Mobile App | BAT, Mindfulness-based therapies |
| [146] | LifeStories | Depression | - | Treatment | Web-based | ACT |
| [147] | MoodGYM | Depression | - | Treatment | Web-based | CBT |
|  | BluePages | Depression | - | Education | - | - |
| [148] | Moodbuster | Depression | - | Education, Treatment | Mobile App, Web-based | CBT, EMA |
| [149] | Supporting Our Valued Adolescents (SOVA) | Anxiety, Depression | - | Treatment | Web-based | - |
| [150] | Living to the Full | Depression | - | Education, Prevention | Web-based | - |
| [151] | PratenOnline | Depression | - | Treatment, Support | Web-based, Text messaging | - |
| [152] | GET.ON Mood Enhancer | Depression | - | Treatment | Web-based | Behavioral therapy and PST |
| [153] | Deprexis | Depression | - | Treatment | Web-based | - |
| [154] | The Internet intervention from the ODIN program | Depression | - | Education, Treatment | Web-based | CT |
| [155] | BioBase program and BioBeams wearable device | Anxiety | - | Education, Monitoring | Mobile app, Wearable technology | - |
| [156] | EVO | Depression | - | Treatment | Mobile App | Gamification |
|  | iPST | Depression | - | Treatment | Mobile App | PST |
| [157] | Alles Onder Controle - Turkish Version (AOC-TK) | Depression | - | Treatment | Web-based | PST |
| [158] | Headgear | Depression | - | Education, Treatment | Mobile App | BA, Mindfulness therapy |
| [159] | Space from Depression | Depression | - | Education, Treatment | Web-based | CBT |
|  | Space from Anxiety | Anxiety | - | Education, treatment | Web-based | CBT |
| [160] | aware | Anxiety | Apple App Store, Google Play Store | Treatment | Mobile App | - |
| [161] | MoodMission | Anxiety, Depression | - | Treatment | Mobile App | CBT |
| [162] | VitalSign6 | Depression | - | Diagnosis, Treatment, Support | Web-based | - |
| [163) | MoodHwb | Depression | - | Education, Treatment | Mobile App, Web-based | Psychoeducation, CBT, Positive psychology, Interpersonal, family systems and behavioral change theories |
| [164] | Good Life Compass | Depression | - | Treatment, Support | Web-based, Text messaging | ACT |
| [165] | Flowy | Anxiety | - | Treatment | Mobile App | Gamification |
| [166] | moodManager | Depression | - | Treatment | Web-based | CBT |
|  | TeleCoaching | Depression | - | Support | - | - |
| [167] | Be Good to Yourself | Depression | - | Treatment | Mobile App | CBT |
| [168] | myCompass | Anxiety, Depression | - | Monitoring, Treatment, Support | Web-based | - |
| [169] | EpxDepression | Depression | - | Monitoring | - | - |
| [170] | eMotion | Depression | - | Education, treatment | Web-based | BA, Physical activity |
| [171] | MoodGYM | Depression | - | Prevention, Treatment | Web-based | CBT |
| [172] | The web-based communication system in the STAR*D project | Depression | - | Support | Web-based, Automated email system | - |
| [173] | Tess | Anxiety, Depression | - | Treatment, Support | AI Chatbot | CBT, Mindfulness-based therapy, Emotionally focused therapy, ACT, Motivational interviewing, Self-compassion therapy, Interpersonal psychotherapy |
| [174] | Mobile Sensing and Support (MOSS) | Depression | - | Monitoring, Treatment | Mobile App | CBT |
| [175] | Help4Mood | Depression | - | Monitoring, Treatment | - | - |
| [176] | Supportive Monitoring and Depression Management over the Internet (SUMMIT) | Depression | - | Monitoring, Treatment | Web-based | - |
| [177] | Psychologist in a Pocket (PiaP) | Depression | - | Monitoring | Mobile App | Experience Sampling Technique |
| [178] | Mindful Moods | Depression | Apple App Store, Google Play Store | Monitoring | Mobile App | EMA |
| [179] | IntelliCare | Anxiety, Depression | Google Play Store | Treatment | - | - |
| [180] | MoodHwb | Depression | - | Education | Web-based | Psychoeducation |
| [181] | Kokoro-App | Depression | - | Treatment | Web-based, Mobile App | CBT |
| [182] | HealthySMS | Depression | - | Monitoring, Support | - | - |
| [183] | E-couch | Anxiety | - | Education, Prevention | Web-based | - |
| [184] | Man Central | Depression | - | Monitoring, Treatment | Mobile App, Web-based | CBT, PST |
|  | myCompass | Depression | - | Education, treatment | Web-based | - |
| [185] | mobiletype | Depression | - | Monitoring | - | Emotional self-awareness |
| [186] | Master Your Mood (MYM) | Depression | - | Treatment | Web-based | CBT |
| [187] | Komuniti Sihat | Anxiety, Depression | - | Education, Treatment | Web-based | Psychoeducation |
| [188] | Panoply | Depression | - | Treatment, Support | Web-based | - |
| [189] | Kurashi-app | Depression | - | Monitoring | Mobile App, Wearable technology | - |
| [190] | Vright | Depression | - | Education, Treatment | Virtual reality software, Wearable technology | Psychoeducation |
| [191] | Depis.Net | Depression | - | Treatment, Support | Web-based | - |
| [192] | Psycho-Babble | Depression | - | Treatment, Support | Web-based | - |
| [193] | Wysa | Depression | Apple App Store, Google Play Store | Treatment, Support | Mobile App | AI chatbot |
| [194] | SituMan | Depression | - | Monitoring | Mobile App | - |
|  | Moodbuster | Depression | - | Monitoring | Mobile App | Ecological Momentary Assessment, Ecological Momentary Intervention |
| [195] | E-Couch | Anxiety | - | Education, Treatment | Web-based | - |
| [196] | Supporting Our Valued Adolescents (SOVA) | Anxiety, Depression | - | Diagnostic, Treatment | Web-based | - |
| [197] | ¡Aptívate! | Depression | - | Treatment | - | BA |
|  | iCouch | Anxiety, Depression | - | Treatment | - | CBT |
| [198] | Mindful Mood Balance | Depression | - | Education, Treatment | Web-based | - |
| [199] | Todac Todac app | Depression | - | Treatment | Mobile App | CBT |
| [200] | Challenger | Anxiety | - | Education, Treatment | Mobile App | - |
| [201] | Happy@Work | Depression | - | Education, Treatment | Web-based, Text messaging | PST, CT |
| [202] | SPSRS | Depression | - | Treatment | Mobile App | - |
| [203] | Overcome Social Anxiety | Anxiety | - | Treatment | Web-based | CBT |
