# Supplementary material for: Characteristics of Mobile Health Platforms for Depression and Anxiety: Content Analysis Through a Systematic Review of the Literature and Systematic Search of Two App Stores
Source: J Med Internet Res. 2022 Feb 4;24(2):e27388. doi: 10.2196/27388 (PMC8857696; doi:10.2196/27388)
Supplement: Multimedia Appendix 3 [file jmir_v24i2e27388_app3.docx]

**Appendix**

**Appendix 3: Table of brief mHealth platform characteristics from the app stores search**

| **Name of mHealth platform** | **Targeted condition** | **App Store** | **Purpose of platform** | **Type of intervention** | **Scientific research source*** |
| --- | --- | --- | --- | --- | --- |
| Anxiety | Anxiety | Google Play Store | Education | Gamification | - |
| Anxiety & Depression Symptoms | Anxiety, Depression | Google Play Store | Education | - | - |
| Anxiety & Stress Trivia | Anxiety | Google Play Store | Education, Diagnostic | - | - |
| Anxiety and Panic Attacks | Anxiety | Google Play Store | Education | - | - |
| Anxiety Cures - Relieve Stress, Increase Happiness | Anxiety, Depression | Google Play Store | Education, Treatment | Problem-solving | - |
| Anxiety Helper | Anxiety | Google Play Store | Treatment | Problem-solving | - |
| Anxiety Meditation Free App: Anxiety Music | Anxiety, Depression | Google Play Store | Treatment | Audio | - |
| Anxiety NOW - Help for Anxiety & Panic Attacks | Anxiety | Google Play Store | Monitoring, Education | - | - |
| Anxiety Release based on EMDR | Anxiety | Google Play Store | Treatment, Monitoring | EMDR | - |
| Anxiety Relief and Antistress Game | Anxiety | Google Play Store | Treatment | Gamification | - |
| Anxiety Relief Apps Guide | Anxiety | Google Play Store | Education | - | - |
| Anxiety Relief Games & Calming games | Anxiety | Google Play Store | Treatment | Gamification | - |
| Anxiety Relief Hypnosis - Stress, Panic Attacks | Anxiety, Depression | Google Play Store | Treatment | Hypnotherapy | - |
| Anxiety Relief Sounds | Anxiety | Google Play Store | Treatment | Audio | - |
| Anxiety Reliever: Mental Health Support | Anxiety | Google Play Store | Treatment, Support | Audio | - |
| ANXIETY SYMPTOMS & How To Deal With Them | Anxiety | Google Play Store | Education | - | - |
| Anxiety Symptoms | Anxiety | Google Play Store | Education | - | - |
| Anxiety Test | Anxiety | Google Play Store | Diagnostic | - | - |
| Anxiety Tool | Anxiety | Google Play Store | Treatment | Audio, EMDR | - |
| Anxiety Tracker - Stress and Anxiety Log | Anxiety | Google Play Store | Monitoring | - | - |
| Anxiety Treatment | Anxiety | Google Play Store | Education | - | - |
| Anxiety | Anxiety | Google Play Store | Treatment | Problem-solving | - |
| Be Mentally Healthy (Stress and Anxiety Therapy) | Anxiety, Depression | Google Play Store | Treatment, Support | Problem-solving | - |
| Beat Depression | Depression | Google Play Store | Treatment | Gamification | - |
| BeSerene \| Mind Training and Stress Management App | Anxiety, Depression | Google Play Store | Prevention, Treatment | Physical activity | - |
| Carpe Diem - Depression and Anxiety Forum | Anxiety, Depression | Google Play Store | Treatment, Support | Forum | - |
| CBT Buddy : Anxiety & Stress | Anxiety | Google Play Store | Treatment | CBT | - |
| CESD Depression Test | Depression | Google Play Store | Diagnostic | - | - |
| Control and Monitor: Anxiety, Mood and Self-Esteem | Anxiety, Depression | Google Play Store | Monitoring | - | - |
| Counseling - Talk, Chat & Video Conference - Live | Anxiety, Depression | Google Play Store | Treatment | Counselling | - |
| Dealing with Depression | Depression | Google Play Store | Education | - | - |
| Dealing With Depression, Depression Treatment | Depression | Google Play Store | Education | - | - |
| Depression | Depression | Google Play Store | Education | Gamification | - |
| Depression - Mental Health Awareness | Depression | Google Play Store | Education | - | - |
| Depression & Anxiety Self-Test (Africa's Version) | Anxiety, Depression | Google Play Store | Diagnostic | - | - |
| Depression & Bipolar Disorder | Depression | Google Play Store | Education | - | - |
| Depression Awareness | Depression | Google Play Store | Education | - | - |
| Depression CBT Self-Help Guide | Depression | Google Play Store | Education, Treatment | CBT | - |
| Depression Cure Music | Depression | Google Play Store | Treatment | Audio | - |
| Depression Medication Manager (Early Access) | Depression | Google Play Store | Monitoring | Gamification | - |
| Depression Support | Depression | Google Play Store | Treatment, Support | Forum | - |
| Depression Test (1) | Depression | Google Play Store | Diagnostic | - | Kroenke, K., Spitzer, R. L., & Williams, J. B. W. (2001). The PHQ-9: Validity of a brief depression severity measure. J Gen Intern Med, 16, 606-613. |
| Depression Test (2) | Depression | Google Play Store | Diagnostic | - | Spitzer RL et al., Validation and utility of a self-report version of PRIME-MD: the PHQ primary care study. Primary Care Evaluation of Mental Disorders. Patient Health Questionnaire.JAMA. 1999 Nov 10;282(18):1737-44.  J Kroenke K et al. The PHQ-9: validity of a brief depression severity measure. Gen Intern Med. 2001 Sep;16(9):606-13. |
| DEPRESSION TREATMENT | Depression | Google Play Store | Education, Treatment | Problem-solving | - |
| Don't panic - Depression and panic help | Anxiety, Depression | Google Play Store | Prevention, treatment | Problem-solving | - |
| Ease My Stress & Anxiety | Depression | Google Play Store | Treatment | Breathing, Meditation | - |
| Easy Psychiatry : Online Clinic, Affirmations | Anxiety, Depression | Google Play Store | Education, Treatment | Forum, Positive psychology, Referral to care | - |
| FearTools - Anxiety Aid | Anxiety | Google Play Store | Treatment, education, Diagnostic | CBT | - |
| FeelJoy - Mental Well-Being for Anxiety | Depression | Google Play Store | Treatment, Education, Support | Forum, Referral to care | - |
| Fight Depression | Anxiety, Depression | Google Play Store | Education, Treatment | Positive psychology, Spiritual health | - |
| Fight Depression Naturally | Depression | Google Play Store | Prevention, Treatment | Recommendations | - |
| Geriatric Depression Scale 2.0 | Depression | Google Play Store | Diagnostic | - | - |
| GPS Mental Health | Anxiety, Depression | Google Play Store | Treatment | Referral to care | - |
| HADS | Depression | Google Play Store | Diagnostic | - | - |
| HeadUp - Anonymous stories and mental health tests | Anxiety, Depression | Google Play Store | Diagnostic, Treatment, Support | Forum | - |
| HearMe \| Empathy Not Therapy | Anxiety, Depression | Google Play Store | treatment | Referral to care | - |
| How To Beat Depression | Depression | Google Play Store | Education, Treatment | Problem-solving | - |
| How To Deal With Anxiety - Anxiety Treatment | Anxiety | Google Play Store | Education, Treatment | Problem-solving | - |
| How to Deal With Anxiety | Anxiety | Google Play Store | Education, Treatment | Problem-solving | - |
| How to Get Over Depression | Depression | Google Play Store | Prevention, Education, Treatment | Problem-solving | - |
| How to Overcome Anxiety | Anxiety | Google Play Store | Education, Treatment | Problem-solving | - |
| How To Overcome Depression | Depression | Google Play Store | Treatment | Problem-solving | - |
| How to Reduce Stress and Anxiety | Anxiety | Google Play Store | Education, Treatment | Problem-solving | - |
| How to Relieve Stress and Anxiety | Anxiety | Google Play Store | Education, Treatment | Problem-solving | - |
| How to Relieve Stress and Anxiety | Anxiety | Google Play Store | Education, Treatment | Problem-solving | - |
| How To Stop Anxiety Attack | Anxiety | Google Play Store | Education, Treatment | Problem-solving | - |
| Hypnosis for Anxiety,Stress and Depression Guide | Anxiety, Depression | Google Play Store | Treatment | Hypnosis | - |
| InnerHour Self-Care Therapy - Anxiety & Depression | Anxiety, Depression | Google Play Store | Education, Treatment, Support | CBT, Positive psychology, Mindfulness, Referral to care | - |
| Insight: For Stress & Anxiety | Anxiety | Google Play Store | Education, Treatment | Problem-solving | - |
| Managing your stress & anxiety | Anxiety | Google Play Store | Education, Treatment | Recommendations | - |
| Mantra - Daily Affirmations, Anxiety, Self Care | Anxiety, Depression | Google Play Store | Treatment | Audio, Positive psychology | - |
| Meditation for Anxiety and Stress | Anxiety | Google Play Store | Treatment | Meditation | - |
| Mental Health Guide | Anxiety, Depression | Google Play Store | Education | - | - |
| Mental Health Recovery Guide | Depression | Google Play Store | Education, Treatment, Support | Recommendations, Positive psychology | - |
| Mental Health Tanzania App | Anxiety, Depression | Google Play Store | Prevention, Treatment, Support | Referral to care | - |
| Mental Health Test (1) | Anxiety, Depression | Google Play Store | Diagnostic | - | - |
| Mental Health Test (2) | Anxiety, Depression | Google Play Store | Diagnostic, Monitoring, Treatment | Referral to care | - |
| Mind Ease: Anxiety Relief | Anxiety | Google Play Store | Treatment | Audio, Problem solving | - |
| Mindfulness For Depression | Depression | Google Play Store | Treatment | Meditation | - |
| Mood Tracker, Journal, Diary \| Anti Depression app | Depression | Google Play Store | Monitoring | - | - |
| Moodily - Mood Tracker, Depression Support | Anxiety, Depression | Google Play Store | Monitoring | - | - |
| My Safe Zone - Anxiety Attack Assistance | Anxiety | Google Play Store | Treatment | Problem-solving, positive psychology | - |
| Online Therapy, Emotional diary, Mindfulness tools | Anxiety, Depression | Google Play Store | Treatment, Support | Referral to care | - |
| Online Therapy: Chat with a Live Therapist | Anxiety, Depression | Google Play Store | Treatment | Referral to care | - |
| Overcome Anxiety | Anxiety | Google Play Store | Treatment | Audio, Positive psychology | - |
| Overcome Depression - How to Cope with Depression | Depression | Google Play Store | Education, Treatment | Problem-solving | - |
| Overcoming Depression | Depression | Google Play Store | Education, Treatment | Problem-solving | - |
| Panic Diary: a anxiety tracker app | Anxiety | Google Play Store | Monitoring | - | - |
| Panic Relief- stop anxiety now | Anxiety | Google Play Store | Treatment, Support | Problem-solving, CBT | - |
| PerSoNClinic ( Depression,Chronic Pain, Cancer) | Depression | Google Play Store | Monitoring | - | - |
| PinkyMind: Online counselling, Anxiety, Depression | Anxiety, Depression | Google Play Store | Treatment, Support | Referral to care | - |
| PsyCare - mental wellness and healthcare | Anxiety, Depression | Google Play Store | Treatment, Support | Referral to care | - |
| PsychApp – Depression, Anxiety, Panic Attack | Anxiety, Depression | Google Play Store | Education, Diagnostic | - | - |
| Psychiatry Pro-Diagnosis,Info,Treatment,CBT & DBT | Anxiety, Depression | Google Play Store | Diagnostic, Treatment, Support | CBT, DBT, Forum | - |
| Qi Gong for Anxiety | Anxiety | Google Play Store | Treatment | Physical activity | - |
| Reduce Depression | Depression | Google Play Store | Education, Treatment | Problem solving | - |
| rTribe - Coaching for Anxiety/Addiction/Depression | anxiety, depression | Google Play Store | Treatment | Referral to care, Forum | - |
| Serenita - Stress & Anxiety | Anxiety | Google Play Store | Treatment | Breathing, Meditation | - |
| Social Anxiety Disorder | Anxiety | Google Play Store | Education | - | - |
| Social Anxiety Test | Anxiety | Google Play Store | Diagnostic | - | - |
| Sooma Depression Therapy Pro | Depression | Google Play Store | Monitoring, Treatment | tDCS | - |
| Stop Anxiety with Relax! | Anxiety | Google Play Store | Treatment | Positive psychology | - |
| STOP Depression | Depression | Google Play Store | Diagnostic | - | - |
| Stress & Anxiety Companion | Anxiety | Google Play Store | Treatment | CBT | - |
| stress relief - stress and anxiety | Anxiety | Google Play Store | Education, Treatment | CBT, Recommendations | - |
| Stress Relief Management Yoga – Anxiety Relief | Anxiety | Google Play Store | Prevention, Monitoring, Treatment, Support | Recommendations | - |
| The Szondi Test: Research of Depression | Depression | Google Play Store | Diagnostic | - | - |
| Therapeer: Peer Emotional Support | Anxiety, Depression | Google Play Store | Treatment | Forum | - |
| Therapy - Professional Mental Health Sessions | Anxiety, Depression | Google Play Store | Treatment, Support | Referral to care | - |
| TherapyChat - Online therapy & counselling | Anxiety, Depression | Google Play Store | Treatment, Support | Referral to care, CBT | - |
| ThinkRight.me: Meditate, Think Positive & Succeed | Anxiety | Google Play Store | Treatment | Meditation, Positive psychology, Recommendations, Audio | - |
| Unwinding Anxiety® | Anxiety | Google Play Store | Treatment, Support | Problem solving | - |
| Wandr. - Stress, Anxiety & Panic Relief | Anxiety | Google Play Store | treatment | Virtual reality, Audio | - |
| Wellness Hub: Video Counselling app for Depression | Anxiety, Depression | Google Play Store | Treatment, Support | Referral to care | - |
| Yoga for Relief of Anxiety, Stress and Depression | Depression | Google Play Store | Treatment | Recommendations | - |
| Depression Test (3) | Depression | Google Play Store | Diagnostic | - | - |
| Depression Test (4) | Depression | Google Play Store | Diagnostic | - | - |
| Depression Screening Tool: PHQ-9 Test | Depression | Google Play Store | Diagnostic | - | - |
| AntiStress Anxiety Relief Game | Anxiety | Apple App Store | Treatment | Gamification | - |
| Anxiety Free: iCan Hypnosis | Anxiety | Apple App Store | Treatment | Hypnotherapy | - |
| Anxiety Reliever:Mental Health Support | Anxiety | Apple App Store | Treatment | Audio, Positive psychology, Recommendations | - |
| Anxiety Screening Test | Anxiety | Apple App Store | Diagnostic | - | - |
| Anxiety Test & Relief | Anxiety | Apple App Store | Diagnostic, Treatment, Support | Referral to care | - |
| anxietyhelper | Anxiety | Apple App Store | Education, Treatment | Referral to care | - |
| Awning - Anxiety Relief | Anxiety | Apple App Store | Treatment | Meditation | - |
| Anxiety Solution: Calmer You | Anxiety, Depression | Apple App Store | Treatment | CBT, Meditation | - |
| Geriatric Depression Scale | Depression | Apple App Store | Diagnostic | - | - |
| Depression relief Help of self hypnosis subliminal | Depression | Apple App Store | Treatment | Positive psychology | - |
| Depression Test and Training | Depression | Apple App Store | Diagnostic, Treatment | Gamification | - |
| Depression Test ByPocketShrink | Depression | Apple App Store | Diagnostic | - | - |
| Depression ZX | Depression | Apple App Store | Treatment | TBSW frequencies | - |
| Doctor On Demand | Anxiety, Depression | Apple App Store | Preventive, Treatment, Support | Referral to care | - |
| Geriatric Depression Scale 15 | Depression | Apple App Store | Diagnostic | - | - |
| Get Rid of Depression with Acupressure Massage Points | Depression | Apple App Store | Treatment | Recommendations | - |
| Ginger Emotional Support | Anxiety, Depression | Apple App Store | Treatment, Support | Referral to care, Physical activity | - |
| Happy Boost, Depression Help | Depression | Apple App Store | Treatment | Hypnotherapy, Meditation | - |
| Healthpiper - Psychiatrist Chat | Anxiety, Depression | Apple App Store | Treatment | Referal to care | - |
| iPrevail | Anxiety, Depression | Apple App Store | Treatment, Support | Forum, Counselling | - |
| Kintsugi | Anxiety, Depression | Apple App Store | Treatment, Monitoring, Support | Counselling, Positive psychology | - |
| Lifehelp - Online Therapy | Anxiety, Depression | Apple App Store | Treatment | Counselling | - |
| Lyf - You're not alone | Anxiety, Depression | Apple App Store | Monitoring, Treatment, Support | Forum, Referral to care | - |
| Mental Health Tests | Anxiety | Apple App Store | Diagnostic | - | - |
| MoodLinks: Anxiety.Depression | Anxiety, Depression | Apple App Store | Treatment | CBT, DBT, Meditation | - |
| Natural Anxiety & Panic Relief | Anxiety | Apple App Store | Treatment | Receommndations | - |
| New! Depression Test | Depression | Apple App Store | Diagnostic | - | - |
| notOK® | Anxiety, Depression | Apple App Store | Treatment | Referral to care | - |
| Panic Attacks or Anxiety? | Anxiety | Apple App Store | Education, Treatment | Meditation | - |
| PHQ-9 Depression Test Questionaire | Depression | Apple App Store | Diagnostic, Monitoring | - | - |
| Rapid Diagnosis- Mental Health | Anxiety | Apple App Store | Diagnostic, Education | - | - |
| Self-manage Depression - GGDE | Anxiety | Apple App Store | Treatment | Positive psychology, CBT | - |
| Simple Depression Test | Depression | Apple App Store | Diagnostic, Monitoring | - | - |
| Switch App | Anxiety | Apple App Store | Treatment | Recommendations | - |
| TalkLife: Depression & Anxiety | Anxiety, Depression | Apple App Store | Treatment, Support | Forum, Referral to care | - |
| Talkspace Online Therapy | Anxiety, Depression | Apple App Store | Treatment, Monitoring, Support | Referral to care | - |
| The Anxiety Guy Audio Podcasts | Anxiety | Apple App Store | Treatment | Problem-solving | - |
| UpLift - Depression & Anxiety | Anxiety, Depression | Apple App Store | Treatment | CBT | - |
| Relax Lite: Stress and Anxiety Relief | Anxiety | Apple App Store | Treatment | Breathing, Meditation | - |
| BetterHelp: Online Counseling & Therapy | Anxiety, Depression | Apple App Store, Google Play Store | Treatment | CBT, DBT, Referral to care | - |
| WeAreMore: Therapy Finder + Free Peer Support App | Anxiety, Depression | Apple App Store, Google Play Store | Treatment | Forum, Referral to care | - |
| Youper- Feel your best | Anxiety, Depression | Apple App Store, Google Play Store | Treatment | ACT, CBT, DBT, Mindfulness | - |
| Dare - Break Free From Anxiety | Anxiety | Apple App Store, Google Play Store | Treatment, Monitoring | Problem-solving | - |
| Aurum | Anxiety, Depression | Apple App Store, Google Play Store | Treatment, Support | CBT, Referral to care | - |
| Flow - Depression | Depression | Apple App Store, Google Play Store | Prevention, Education, Diagnostic, Treatment | Physical exercise, Meditation, Recommendations, tDCS (only when used with the Flow headset) | - |
| Happy - A Mental Health App for everyone | Anxiety, Depression | Apple App Store, Google Play Store | Monitoring, Treatment | Audio, Breathing, Recommendations, Positive psychology | - |
| Lift – Depression & Anxiety | Anxiety | Apple App Store, Google Play Store | Education, Monitoring, Treatment | Forum | - |
| MindShift CBT - Anxiety Canada | Anxiety | Apple App Store, Google Play Store | Monitoring, Treatment | CBT | - |
| Moodpath - Depression & Anxiety Test | Anxiety | Apple App Store, Google Play Store | Education, Monitoring, Diagnostic | - | - |
| MoodTools - Depression Aid | Depression | Apple App Store, Google Play Store | Education, Monitoring, Diagnostic | CBT, BAT, Recommendations | - |
| Pocketcoach - Anxiety Helper | Anxiety | Apple App Store, Google Play Store | Education, Treatment, Support | CBT, ACT | - |
| Rootd - Panic Attack & Anxiety Relief | Anxiety | Apple App Store, Google Play Store | Education, Treatment, Support | Meditation, Breathing, Recommendations, Referal to care | - |
| Sanvello for Stress, Anxiety & Depression | Anxiety, Depression | Apple App Store, Google Play Store | Monitoring, Treatment, Support | Counselling (only in US), Forum, CBT, Recommendations, Referral to care | - |
| Shine: Calm Anxiety & Sleep | Anxiety | Apple App Store, Google Play Store | Treatment, Support | Positive psychology, Meditation, Audio, Forum | - |
| Wysa: stress, depression & anxiety therapy chatbot | Anxiety, Depression | Apple App Store, Google Play Store | Monitoring, Treatment, Support | Counselling, CBT, DBT, Meditation, Physical activity | - |
| Relax: Stress & Anxiety Relief / Relax Lite: Stress & Anxiety Relief | Anxiety | Apple App Store, Google Play Store | Treatment | Meditation | - |
| At Ease Anxiety & Worry Relief | Anxiety | Apple App Store, Google Play Store | Treatment | Meditation | - |
| BetterHelp - Online Counseling | Anxiety, Depression | Apple App Store, Google Play Store | Treatment | Referral to care | - |
| Self-help for Anxiety Management (Apple) Self-help Anxiety Management (Google) | Anxiety | Apple App Store, Google Play Store | Monitoring, Treatment | Problem-solving, Breathing | - |
| 7 Cups: Anxiety & Stress Chat | Anxiety | Apple App Store, Google Play Store | Treatment, Support | Counselling, Recommendations, Forum | - |
| MoodMission - Cope with Stress, Moods & Anxiety | Anxiety | Apple App Store, Google Play Store | Treatment | CBT, Meditation, Recommendations, Positive Psychology | Bakker, D., Kazantzis, N., Rickwood, D., & Rickard, N. (2018). Development and pilot evaluation of smartphone-delivered cognitive behavior therapy strategies for mood-and anxiety-related problems: MoodMission. Cognitive and Behavioral Practice, 25(4), 496-514.  Bakker, D., & Rickard, N. (2019). Engagement with a cognitive behavioural therapy mobile phone app predicts changes in mental health and wellbeing: MoodMission. Australian Psychologist, 54(4), 245-260. |

*References were extracted from app store descriptions
